# Unusual Presentation of a Rare Disease: A Case Report of Fungal Necrotizing Otitis Media and Mastoiditis

**DOI:** 10.1002/ccr3.70101

**Published:** 2025-01-20

**Authors:** Ermia Mousavi Mohammadi, Mohsen Rajati, Hossein Zarrinfar

**Affiliations:** ^1^ Department of Otorhinolaryngology, Sinus and Surgical Endoscopic Research Center, School of Medicine Mashhad University of Medical Sciences Mashhad Iran; ^2^ Allergy Research Center Mashhad University of Medical Sciences Mashhad Iran

**Keywords:** *Aspergillus*, *Candida*, fungal, necrotizing, otitis media

## Abstract

Acute necrotizing otitis media is a severe middle ear infection which causes necrosis of the tympanic cavity. A 54‐year‐old female was presented who suffered from diabetes mellitus and end‐stage renal disease presenting with severe otalgia, initially thought to be necrotizing otitis externa. She rapidly progressed to total necrosis of the tympanic membrane. Further assessments confirmed a fungal disease caused by *Candida tropicalis* and *Aspergillus flavus*. Immediate antifungal therapy with amphotericin B and subsequent radical mastoidectomy managed to control the disease with no sign of necrosis after 3 months. Early diagnosis, aggressive systemic antifungal treatment, and surgical debridement are vital components for successful management of such cases.

## Graphical abstract

An unexpected presentation of a rare middle ear disease, fungal acute necrotizing otitis media, has been reported. Although there is no agreed approach in the literature, the way to early diagnosis and, in our opinion, correct management of this rare disease is described.

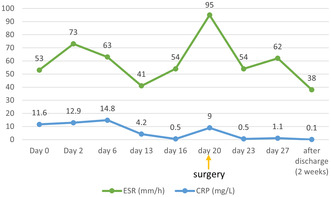

**Summary**

- Given its potential for atypical presentation, it is imperative to maintain a high index of suspicion and actively investigate for this condition, utilizing paraclinical data to aid in early diagnosis.

## 1 Introduction

Otitis media (OM) encompasses a spectrum of inflammatory conditions affecting the middle ear, including acute, recurrent, chronic otitis media, otitis media with effusion (OME), acute necrotizing otitis media (ANOM), and cholesteatoma [1]. Characterized by severe middle ear infection leading to tympanic cavity necrosis, ANOM poses a significant challenge due to its severity, comparative rarity and lack of precise prevalence data [2]. ANOM is frequently observed in individuals with compromised immune systems [3]. While bacterial infections are the primary cause of ANOM, fungal infections are more common in the external auditory canal. Instances of invasive fungal infections originating from the mastoid and middle ear are infrequent [4]. The absence of specific diagnostic criteria for ANOM, particularly regarding fungal agents, further complicates its management, highlighting the need for a well‐defined protocol. This study reported a case of fungal ANOM and discussed its symptoms and management.

## 2 Case History

A 54‐year‐old female, with a significant medical history for diabetes mellitus, hypertension, and end‐stage renal disease (ESRD) referred to the otolaryngology clinic with intermittent right‐sided ear pain for 3 months. The pain was described as dull and localized, worsening at nights, with a severity rating of 8 out of 10, accompanied by gradual deteriorating of hearing and non‐pulsatile tinnitus over the past few days. The patient had no complaint of active ear discharge, facial nerve palsy, or any nasal issues.

Physical examination revealed stable vital signs and a patent external ear canal with mild edema in the right ear, along with dark‐red opacification of the intact tympanic membrane, obscuring landmarks. No discharge or granulation tissues were noticed. Tuning fork examination confirmed right‐sided conductive hearing loss. The patient was provisionally diagnosed with possible necrotizing otitis externa (NOE) and admitted for IV antibiotics and further evaluation.

**FIGURE 1:**
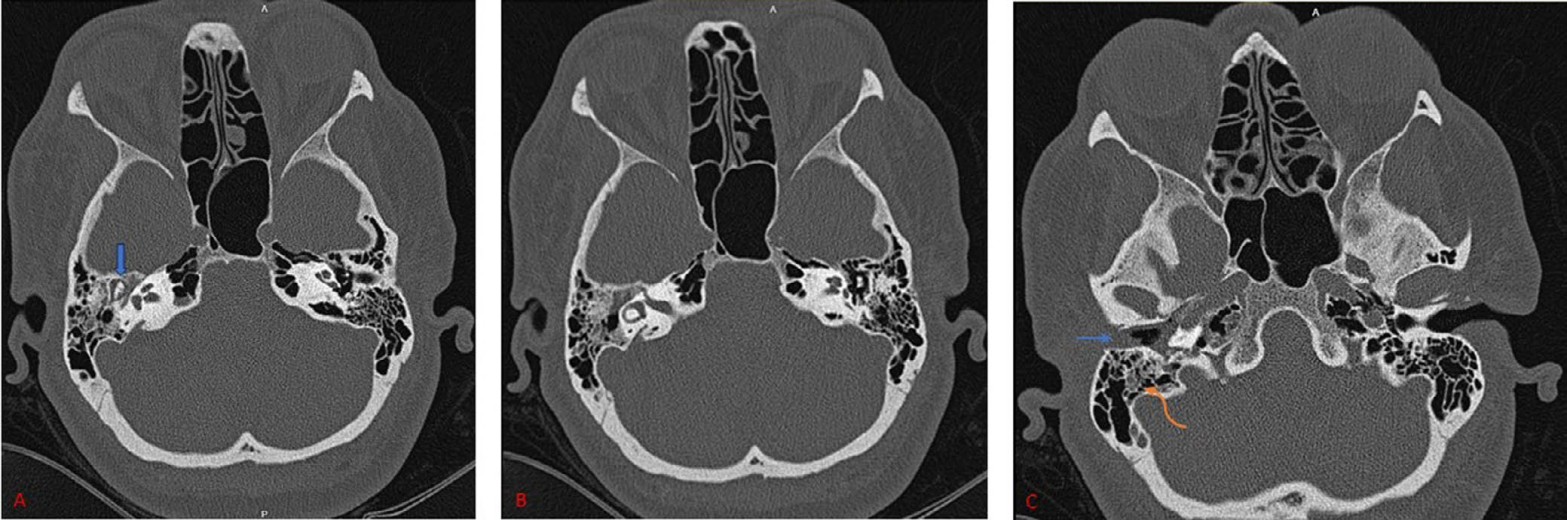
Axial temporal bone computed tomography (CT) scan. (A) Opacification in the attic (blue arrow). (B) Antrum is also opacified. (C) Few mastoid air cells have opacification (curve arrow), soft tissue density without any erosion in bony canal is seen (blue arrow), that was cerumen.

**FIGURE 2:**
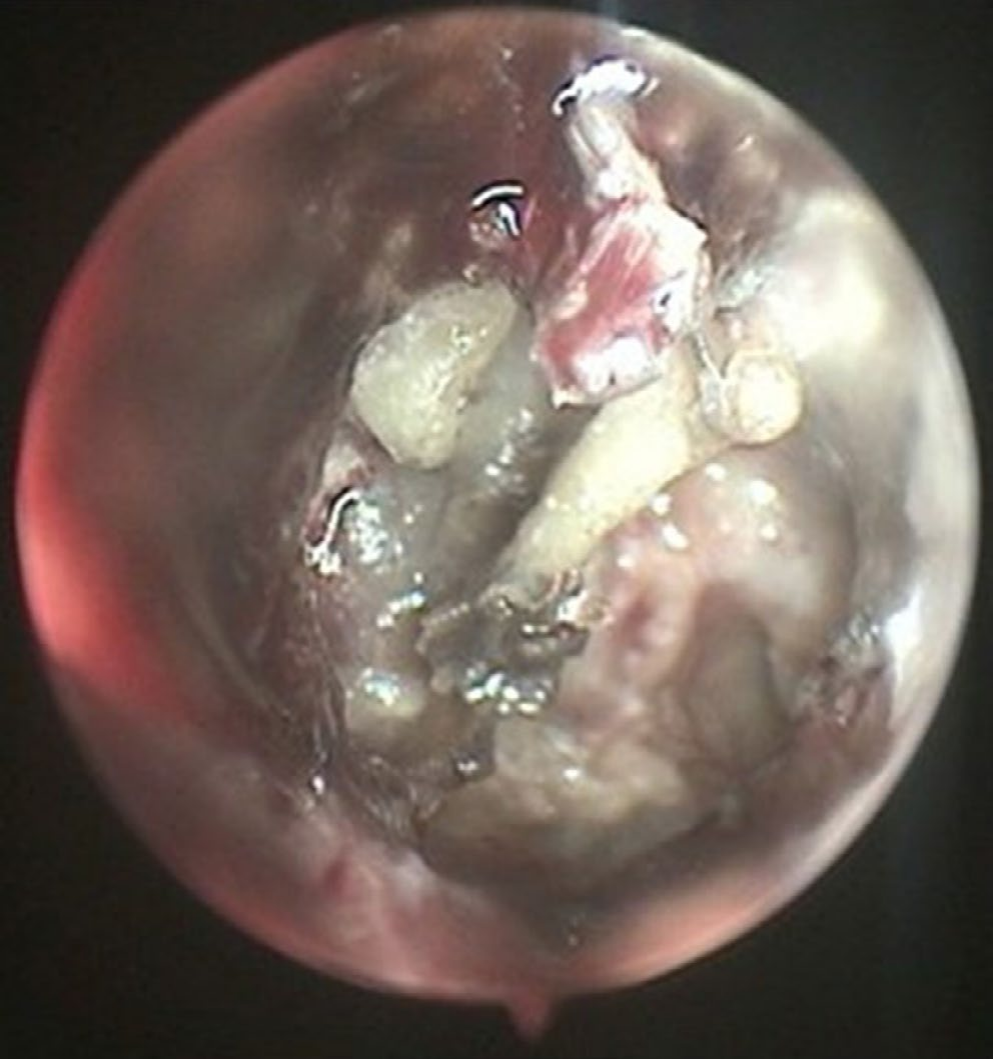
Trans canal endoscopic view of tympanic membrane (TM) shows TM necrosis. Malleolus and tympanic cavity are apparent.

**FIGURE 3:**
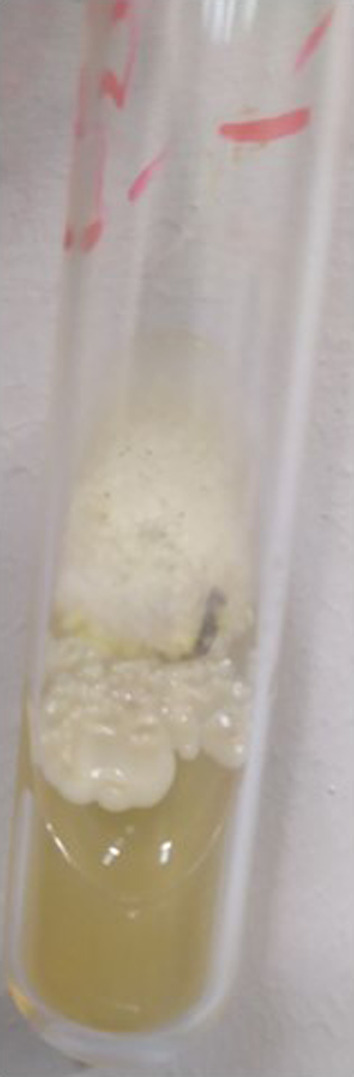
Colonies of *Candida tropicalis* /*Aspergillus flavus* in Sabouraud dextrose agar culture medium related to primary middle ear biopsy at 35oc.

A pure tone audiogram revealed an air‐bone gap (conductive hearing loss) on the right side. Tympanometry of the right ear was typing B pattern. The bone scan utilizing Technetium‐99 (Tc‐99 m) indicated no signs of osteomyelitis in the skull base. High‐resolution computed tomography of the temporal bone demonstrated opacification in the middle ear and mastoid air cells, without bone erosion (Figure 1). Within few days the ear condition rapidly worsened. Otoscopy on the 4th admission day, revealed total necrosis of the tympanic membrane with black discharge (Figure 2). A sample of the necrotic tissue was obtained and submitted to the medical mycology and pathology laboratories for further work‐up. Direct microscopic examination of clinical specimens in 15% potassium hydroxide (KOH) showed septate, branched, hyaline hyphae along with budding cells. Moreover, the clinical specimen was inoculated on Sabouraud dextrose agar with chloramphenicol followed by incubation at 35°C for 3–5 days. The culture results yielded *Candida* and *Aspergillus* colonies (Figure 3). Molecular method, macroscopic and microscopic characteristics revealed *C. tropicalis* and *A. flavus* , as described previously [5, 6].

**FIGURE 4:**
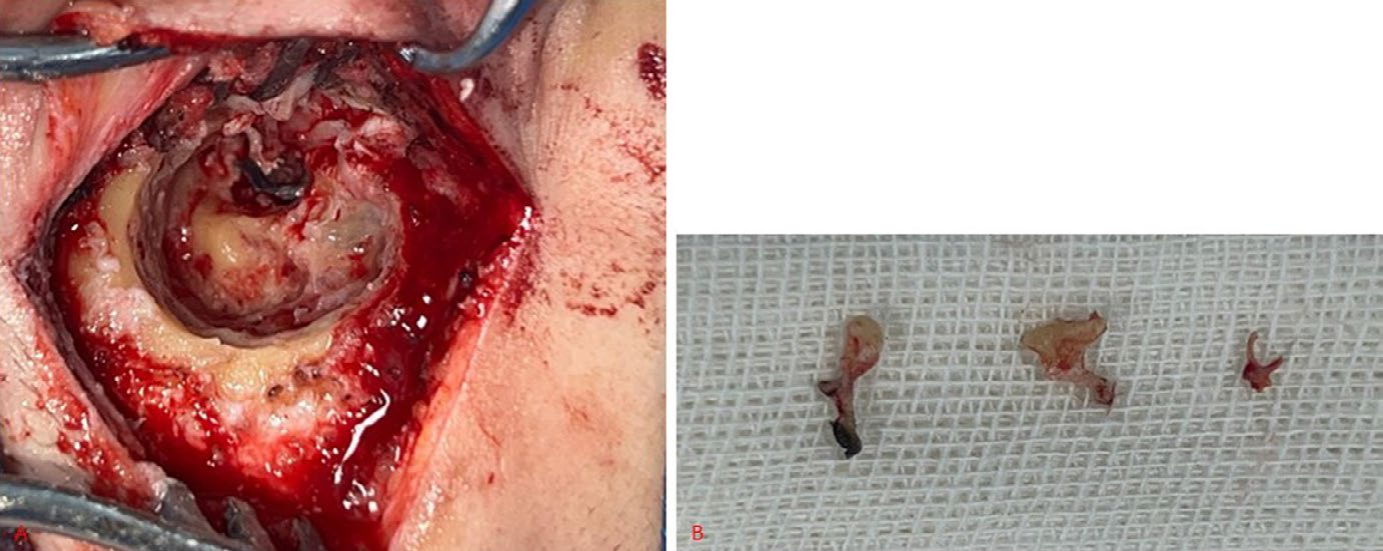
(A) The antrum is opened and lateral semicircular canal is apparent. The black necrotic tissue is seen in the attic. (B) Three ossicles are shown. Handle and lateral process of malleolus, long process of incus, especially lentiform process, and capitulum are necrotic.

Consequently, liposomal amphotericin was added to the treatment regimen and the patient was scheduled for surgical debridement of the ear. Upon opening the mastoid and the antrum, it was observed that the mucosa was necrotic, the attic and mesotympanum mucosa were also abnormal and inflamed. Incus, malleolus and stapes superstructures displayed signs of necrosis, necessitating excision. Foot plate was preserved (Figure 4). The tympanic segment of the facial nerve was dehiscent. A radical mastoidectomy (canal wall down, along with closure of the eustachian tube) was also conducted. We refrained from any surgical intervention at the site due to concerns about a relapsing infectious condition and the need for further follow‐up. Additionally, ossiculoplasty was deemed inappropriate given the limitations associated with a canal wall down procedure, and therefore, it was not performed.

**FIGURE 5:**
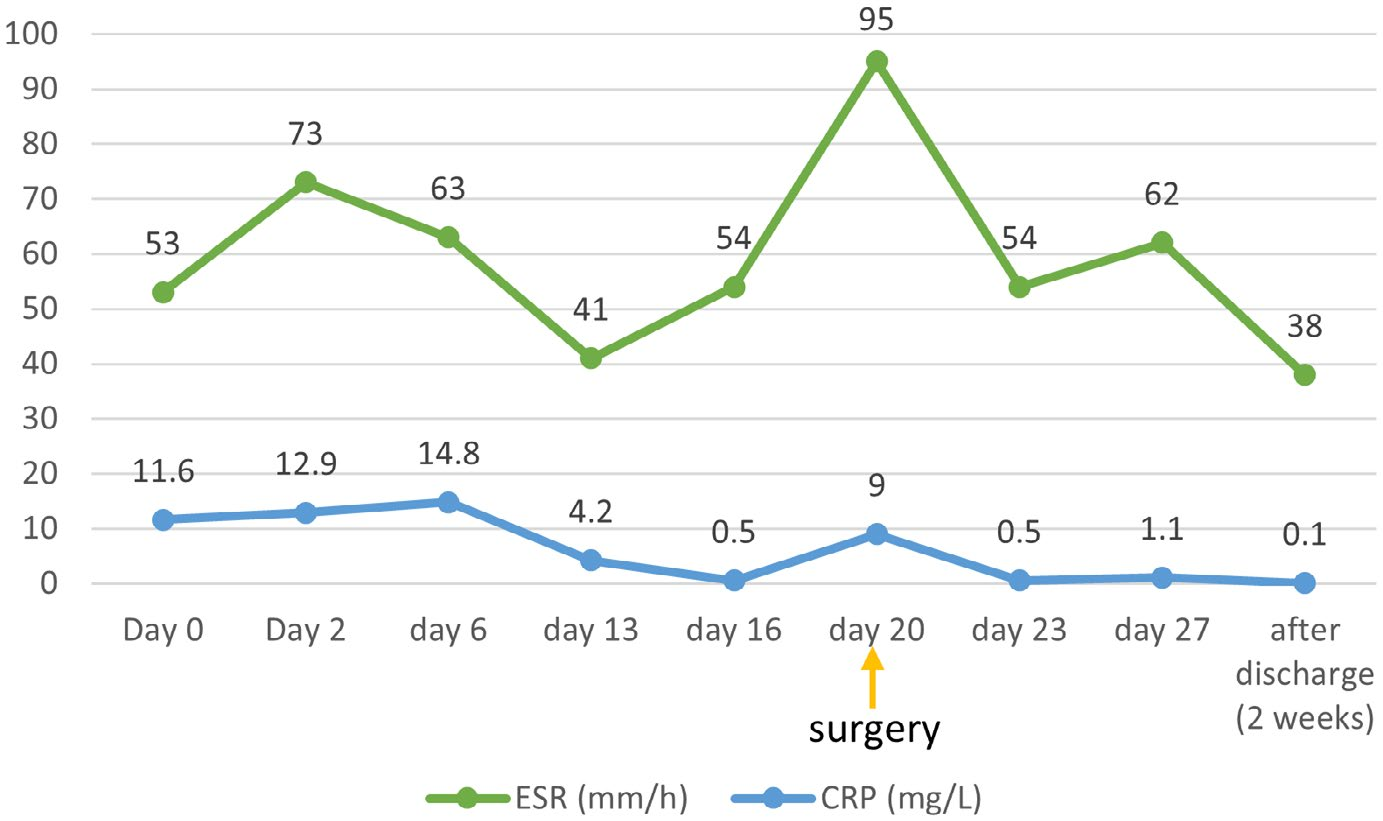
Diagram demonstrated the fluctuation in CRP and ESR levels, from day of admission to 2 weeks after discharge.

Treatment response was regularly monitored with clinical signs and symptoms, erythrocyte sedimentation rate (ESR) and C‐reactive protein (CRP) as depicted in Figure 5. By 3 weeks of IV antifungal treatment, she was free of symptoms, and discharged from the hospital on oral voriconazole to be taken for 20 more days.

## 3 Outcome and Follow Up

On 3 months follow‐up visit, the patient had no complaint of any otologic symptoms except for non‐pulsatile tinnitus and hearing loss on the same ear. The audiogram indicated conductive hearing loss, characterized by a 45 dB air‐bone gap and a notable drop in bone conduction at high frequencies. The speech recognition threshold was measured at 65 dB sound pressure level (SPL). In contrast, the left ear exhibited normal hearing levels, and the patient reported no difficulties in daily conversations. No sign of recurrence was detected on her examination and the mastoid cavity was completely epithelialized.

## 4 Discussion

Invasive aspergillosis (IA) in the middle ear is rare, with limited reported cases in the literature. Liu et al.'s comprehensive review identified only seven cases of invasive mastoiditis or ANOM associated with aspergillosis [7]. ANOM is a rapidly invasive infection, characterized by extensive middle ear necrosis, leading to the destruction of its contents and the tympanic membrane [2, 8]. In this case, initial examination only showed an opacification of the tympanic membrane. However, complete necrosis of the tympanic membrane occurred within 4 days. This presentation raises the possibility of NOE extending into the tympanic cavity; however, subsequent observations suggest that this diagnosis is unlikely.

ANOM particularly affects individuals with compromised immune systems, as seen in our case with concurrent diabetes mellitus and end‐stage renal disease [8]. While bacterial infections, notably beta‐hemolytic *Streptococcus*, are common causes, there is an increasing recognition of IA as an etiological factor for ANOM. Chen et al. proposed a classification system for fungal ear infections, categorizing them based on the extent of inflammation and the presence of facial nerve palsy, aiding in tailored treatment strategies [9] (Table 1).

**TABLE 1:** Classification of fungal infections of the ear and temporal bone by Chen et al. [9].

Type	Extension of the disease	Treatment
I	Limited otitis externa	Cleaning; topical anti‐fungal agents
II	Otitis externa with mastoiditis	Topical and systemic anti‐fungals; surgery for consideration (if no improvement)
III	Invasive mastoiditis with facial nerve palsy	Amphotericin B; itraconazole; surgical debridement
IV	Invasive mastoiditis, facial nerve palsy with otogenic skull base osteomyelitis (SBO)	Amphotericin B; itraconazole; surgical debridement

Regarding diverse structures of the middle ear, symptoms of ANOM vary with otalgia and otorrhea being common, along with conductive hearing loss [3]. Less common, facial palsy can also be observed in patients with ANOM [9].

Physical examination of the ear may suggest that the middle ear cavity may is filled with granulation tissue, with no discernible landmarks, there may be necrosis or perforation of the tympanic membrane [3]. If ANOM is associated with NOE, the auditory canal may be inflamed and swollen, obstructing the view of the tympanic membrane and the structures behind that [10, 11]. This situation may impede or even conceal the identification of ANOM.

In this case, initial presentation lacked typical signs of ANOM, highlighting diagnostic challenges. The rapid progression to tympanic membrane necrosis despite antibiotic treatment raised suspicion of fungal ANOM. Furthermore, the ear pain resolved to a good extent after 4 days of intravenous antibiotic treatment, showing the possibility of additional polymicrobial and bacterial infections. However, rapid progression to tympanic membrane necrosis despite antibiotic treatment brought about the possibility of fungal ANOM.

Elevated ESR despite complete treatment and improvement of symptoms and signs could be relevant to underlying ESRD [12].

Management includes controlling underlying conditions, such as blood glucose levels, surgical removal of infected tissue, and administering antifungal medication. In cases of invasive *Aspergillus* otitis externa, many clinicians suggest a surgical biopsy for early diagnosis. Surgical debridement in the early stages is also recommended for patients who do not respond to conventional antifungal therapy [13, 14]. According to Dominik et al., if empirical treatment is unsuccessful for otogenic skull base osteomyelitis caused by invasive fungal infection, radical mastoidectomy should be performed within 2 weeks [15], similar to invasive fungal rhinosinusitis, in which the mainstay treatment involves radical debridement combined with antifungal therapy.

Upon admission, the immediate focus was on glycemic control. Hemodialysis was conducted three times per week. On the fifth day, after confirming the presence of IA, intravenous antifungal therapy was added to the antibiotic regimen for 2 weeks. Subsequently, a radical mastoidectomy was performed, and the intravenous antifungal therapy was sustained for an additional week. After consulting with an infectious disease specialist, the patient was discharged with a prescription for oral voriconazole to be taken for 20 days. This approach was successful and may serve as a reasonable strategy for similar cases.

The prognosis of invasive fungal OM hinges on several factors, including the patient's immune status, timely diagnosis, and aggressive treatment. Given its potential for atypical presentation, it is imperative to maintain a high index of suspicion and actively investigate for this condition, utilizing paraclinical data to aid in early diagnosis. Our case underscores the effectiveness of a multidisciplinary approach involving radical surgical debridement and full‐dose antifungal therapy with amphotericin B for successful treatment of fungal OM caused by *C. tropicalis* and *A. flavus* . Nevertheless, further cases are warranted to refine and establish the optimal management strategy for similar presentations in the future. Continued research and collaboration within the medical community are essential for further enhancement of our understanding and management of this rare but potentially devastating condition.

## Author Contributions

**Ermia Mousavi Mohammadi:** investigation, writing – original draft. **Mohsen Rajati:** investigation, supervision, writing – review and editing. **Hossein Zarrinfar:** investigation, supervision.

## Ethics Statement

This case (a part of the main project) was approved with an Ethics Committee code: IR.MUMS.fm.REC.1395.121.

## Consent

Written informed consent was obtained from the patient.

## Conflicts of Interest

The authors declare no conflicts of interest.

## Data Availability

The data that support the findings of this study are available from the corresponding author upon reasonable request.
